# Psychometric evaluation of the Mental Health Continuum-Short Form (MHC-SF) in Chinese adolescents – a methodological study

**DOI:** 10.1186/s12955-015-0394-2

**Published:** 2015-12-10

**Authors:** Cheng Guo, Göran Tomson, Jizhi Guo, Xiangyun Li, Christina Keller, Fredrik Söderqvist

**Affiliations:** Medical Management Centre, Department of Learning, Informatics, Management and Ethics, Karolinska Institutet, Stockholm, Sweden; Department of Public Health Sciences, Karolinska Institutet, Stockholm, Sweden; School of Management, Weifang Medical University, Weifang, China; Jönköping University, International Business School, Jönköping, Sweden; Center for Clinical Research, Uppsala University, County hospital, Västerås, Sweden; Competence Center for Health, County Council of Västmanland, Västerås Hospital, Västerås, Sweden

**Keywords:** Mental Health Continuum-Short Form, Chinese adolescents, Reliability, Validity

## Abstract

**Background:**

In epidemiological surveillance of mental health there is good reason to also include scales that measure the presence of well-being rather than merely symptoms of ill health. The Mental Health Continuum-Short Form (MHC-SF) is a self-reported scale to measure emotional, psychological and social well-being and conduct categorical diagnosis of positive mental health. This particular instrument includes the three core components of the World Health Organization’s definition of mental health and had previously not been psychometrically evaluated on adolescents in China.

**Methods:**

In total 5,399 students (51.1 % female) from schools in the urban areas of Weifang in China were included in the study (mean age = 15.13, SD = 1.56). Participants completed a comprehensive questionnaire with several scales, among them the MHC-SF. Statistical analyses to evaluate reliability, structural validity, measurement invariance, presence of floor and ceiling effects and to some extent external validity of the MHC-SF were carried out.

**Results:**

The Cronbach’s α coefficients for sub-scales as well as the total scale were all above 0.80 indicating good reliability. Confirmative factor analysis confirmed the three-dimensional structure of the Chinese version of MHC-SF and supported the configural and metric invariance across gender and age. Noteworthy ceiling effects were observed for single items and sub-scales although not for the total scale. More importantly, observed floor effects were negligible. The stronger correlation found between MHC-SF and Minneapolis-Manchester Quality of Life Instrument (as measure of positive mental health) than between MHC-SF and Hospital Anxiety Depression Scale (as measure of mental illness and distress) yielded support for external validity.

**Conclusion:**

In conclusion, the main findings of this study are in line with studies from other countries that evaluated the psychometric properties of the MHC-SF and show that this instrument, that includes the three core components of the WHO definition of mental health, is useful in assessing positive adolescent mental health also in China.

## Background

In the beginning of the millennium, the burden of mental disorders ranked first over all diseases in China and accounted for approximately 20 % of the total burden of disease [1]. Half of these illnesses begin by the age of 14 and three-quarters by mid-20s [2]. Thus, adolescence is a critical developmental period with long-term implications for the well-being of the individual and for society as a whole [3]. In China, about 175 million adolescents were identified by the latest national population census in 2010 [4]. They experienced the lowest rate of mental health compared to other age groups [5]. By 2005, the prevalence of mental health problems in children and adolescents under the age of 17 years reached 15.6 % [6]. In numbers this equals about 30 million people. Mental health problems may comprise a broad range of mild to severe symptoms, of which the more severe include mental disorders with significant functional impairments as well as adverse effects on life quality.

Patients with mental disorders are a minor group among the whole population but at great medical expense. Accordingly, the focus of mental health research and practice has been on the treatment of pathologies such as depression and anxiety disorders, and to some extent to their prevention. It has been assumed that well-being would prevail when pathology was absent. However, a growing body of evidence shows that high levels of well-being are good for individuals and society and is associated with a range of positive outcomes [7–9]. This is the rational for using measurement instruments that in line with modern definitions of mental health capture more than mental problems and diseases.

According to the World Health Organization mental health is a state of well-being in which the individual realizes his or her own abilities, can cope with the normal stress of life, can work productively and fruitfully, and is able to make a contribution to his or her community [10]. In this positive sense mental health contains three core components – i.e., state of well-being and effective functioning for both the individual and for the community [11] – and builds on two longstanding traditions in studies on life going well [12, 13]. These are the *hedonic* tradition and the *eudaimonic* traditions [14, 15]. Presence of emotional well-being plays an important role in mental health. The *hedonic* tradition, dating from the Greek ages, claims that mental health is determined by feeling and emotion. This approach focuses on happiness and defines well-being as pleasure attainment and negatives avoidance [12]. Subjective well-being consisting of life satisfaction, the presence of positive mood, and the absence of negative mood, refers to happiness and includes emotional well-being as a specific dimension. The limitation of the theory includes neglecting the value and meaning aspect of well-being. The *eudaimonic* tradition, on the other hand, emphasises meaning and self-realization of the individual and relates well-being to the extent that a person is fully functioning [12].

Recently Huppert and So identified ten features of positive well-being, combining hedonic and eudaimonic aspects: competence, emotional stability, engagement, meaning, optimism, positive emotion, positive relationships, resilience, self-esteem and vitality. Based on a psychometric analysis of indicators of these ten features, an operational definition of flourishing was developed using data from a sample of 43,000 Europeans [16]. However, this definition is not fully connected to the WHO definition of mental health, as the society contribution component is missing.

The concept of flourishing has also been raised by Keyes, combining the hedonic and eudaimonic aspects of well-being [17]. To operationalize and measure this concept Keyes developed an instrument, initially labelled as the Mental Health Continuum - Long Form consisting of 40 items [17]. Later the Mental Health Continuum-Short Form (MHC-SF) was adapted from the long form to address the problem with diagnostic threshold and to create a version more efficiently administered in epidemiological surveillance [18]. The MHC-SF consists of items derived from Ryff’s model of psychological well-being [13], Keyes model of social well-being [14] and Bradburn’s affect balance scale [19]. What is particularly interesting with this instrument is that its latent factors mirror all three core components of the WHO definition of mental health [11].

The MHC-SF measures three levels of positive mental health: flourishing, moderate and languishing mental health. Briefly, people who are flourishing in life report having high levels of well-being, meaning that they often experience positive emotions and function well both from a psychological and social perspective. On the other hand, languishing is the absence of mental health as a state of being mentally unhealthy, equivalent to stagnation and emptiness or that life lacks interest and engagement [17]. So far, the MHC-SF has been successfully tested in different countries such as South Africa, Poland, Italy, South Korea and Brazil [20–26] and also shown good psychometric properties on a sample of Chinese adults [27, 28]. Data on the utility of the MHC-SF on adolescents specifically are scarce. One South Korean [20] and one Polish study [21] gave results in line with those previously shown for adults. However, the MHC-SF has not yet been tested on Chinese adolescents. Our hypothesis was that MHC-SF would be useful also in the latter group and to have that confirmed we set out to evaluate the instrument’s psychometric properties. More specifically this evaluation meant checking 1) internal reliability, 2) dimensional structure, 3) invariance across groups and 4) presence of floor and ceiling effects. Further, previous studies have indicated a negative correlation between MHC-SF and anxiety and depression [21] and discussed the need to study the correlation between positive mental health and health-related quality of life [29]. An additional aim of the study was therefore to evaluate external validity of the MHC-SF by correlational analyses with two other scales, one that measures anxiety and depression and one that measures health-related quality of life. We hypothesized that the correlation between the MHC-SF and health-related quality of life as a positive mental health measure would be stronger than the correlation between MHC-SF and a measure of mental illness such as anxiety and depression.

## Methods

### Recruitment and data collection

The research area in China was the city of Weifang in central Shandong Province of P. R. China carrying a population of about 9 million people. Only students who lived in the urban area of Weifang during the data collection period were included. We conducted a stratified and clustered random sampling scheme on Grade 8 in 12 middle schools and Grade 10 in 5 high schools in two unequally socio-economically developed urban districts. Vocational school students were not involved. The sampling frame involved stratification by district and school type, and clustering by school.

A total of 5,399 students from 7 middle schools and high schools were recruited from the urban area of the city of Weifang in April and May 2014. In four of the schools the questionnaires were distributed and completed in the classroom in a given one-hour period and then collected by the researchers. In the other three schools, students were asked to fill in the questionnaires at home and bring them back to the teachers the following day. Students who were absent from school on the day when the questionnaires were distributed were excluded from the study. The response rate was 100 %.

### Measures

#### Mental Health Continuum-Short Form (MHC-SF)

MHC-SF comprises 14 items, representing the three dimensions of well-being. Respondents are asked to rate their feelings in the past month on a 6-point Likert scale (never, once or twice a month, about once a week, two or three times a week, almost every day, every day). Individuals who are diagnosed languishing or flourishing must exhibit low or high levels on at least seven or more of the scales. Individuals are diagnosed as flourishing if they feel 1 of the 3 hedonic well‐being symptoms “every day” or “almost every day” and feel 6 of the 11 positive functioning symptoms “every day” or “almost every day” in the past month. A diagnosis of languishing is made if 1 of the 3 hedonic well‐being symptoms are perceived “never” or “once or twice a month” and 6 of the 11 positive functioning symptoms are perceived “never” or “once or twice a month”. Individuals who are neither “languishing” nor “flourishing” are categorized as “moderately mentally healthy” [30].

#### Minneapolis-Manchester Quality of Life – Adolescent Form (MMQL)

The Minneapolis-Manchester Quality of Life (MMQL) is a self-reported instrument to assess health-related quality of life [31]. The MMQL-Adolescent Form was specially developed for individuals aged 13 to 20. The questionnaire has 46 items comprising the following 7 domains: physical functioning, cognitive functioning, social functioning, outlook on life, intimate relations, psychological functioning and body image. To our knowledge MMQL was used for the first time in China, but it has been tested in some countries such as United States, United Kingdom, Sweden, South Korea and Japan and showed good psychometric properties [31–35]. This instrument was used in the present study to evaluate the external validity of the Chinese version of MHC-SF.

#### Hospital Anxiety and Depression Scale (HADS)

The Hospital Anxiety and Depression Scale (HADS) is a widely used instrument comprising 14 items of psychological distress [36] of which seven measure symptoms of anxiety and seven measure symptoms of depression. It was designed for patients with various diseases but showed high tolerance on general populations [37] and also adolescents [38, 39]. This instrument has also been psychometrically evaluated on Chinese adolescents [39]. In the present study some wording has been slightly changed according to the comments we received in a pilot study on a sample of 285 adolescents.

### Translation procedure

The questionnaire items were translated into Chinese by the research team from China, including the main author and other two Chinese authors and then back translated into English by the main author and a Swedish author. Therefore the two research groups were able to check the quality of translation respectively. To ensure that the items in Chinese were appropriate, we conducted a pilot study on a small group of 285 school students in advance to confirm that the questionnaire in Chinese was readable and acceptable by young adolescents. After this verification, the main study was started.

### Ethical considerations

Ethical approval was acquired from the local administration at Weifang Medical University before the survey study was performed. The headmasters and the directors of student health from all schools were contacted for approval of participation and gave their permission. The informed consent was attached in the introduction part of the questionnaire. Participation was voluntary and each student decided whether to fill in the questionnaire after reading the informed consent. All students who were asked for consent participated in the study and remained anonymous.

### Data analysis

All responses from the survey questionnaires were entered into a computer-based database. SPSS 22 for Windows was used for analyzing the reliability and external validity of MHC-SF. Confirmatory factor analysis was performed by AMOS 22. Internal consistency reliability for MHC-SF was attained by calculating Cronbach’s alpha coefficient. A coefficient greater than 0.70 indicates acceptable reliability, whereas that greater than 0.90 is recommended for better precision [40]. Cronbach’s alpha coefficients for the MHC-SF total scale and each sub-scale were calculated. To confirm the three-factor model, we performed confirmatory factor analysis (CFA) using AMOS 22. Goodness of fit of the CFA models was evaluated with the Root Mean Square Error of Approximation (RMSEA) method [41], the Comparative Fit Index (CFI) [42] and Standardized Root Mean Square Residual (SRMR) [42]. RMSEA values smaller than 0.08 or 0.05 indicate adequate or close fit respectively. CFI values above 0.90 are seen as acceptable values above 0.95 as satisfied. SRMR values close to 0.08 suggest good model fit. The multigroup confirmatory factor analysis was performed to test the measurement invariance across gender and grade groups for the whole sample. For testing loading invariance, a change of ≥ −0.010 in CFI, supplemented by a change of ≥ −0.015 in RMSEA or a change of ≥ −0.030 in SRMR would indicate noninvariance [43]. External validity was assessed comparing the strength of the correlation between measures of positive mental health, i.e., MHC-SF and MMQL, with MHC-SF and HADS as a measure of illness and distress. The strength of these correlations was evaluated using Fisher Z-transformation [44]. Differences between correlated correlation coefficients were tested using the method by Meng et al. [45]. Floor or ceiling effects was considered to be pronounced if more than 15 % of respondents achieved the lowest or highest possible score, respectively [46].

## Results

### Characteristics of subjects

51.1 % of participants were female. The mean age (SD) of all respondents was 15.13 (1.56) in the age range of 11–19 years. The mean ages (SD) of 3,044 participants from Grade 8 and 2,355 participants from Grade 10 are 14.34 (1.58) and 16.18 (0.64), respectively.

### Reliability

The Cronbach’s alpha coefficient for the overall scale (α = 0.92) indicated high internal consistency for the total MHC-SF. Coefficients for the subscales of emotional well-being (α = 0.92), social well-being (α = 0.83) and psychological well-being (α = 0.86) were all considered to be satisfactory.

### Structural validity

**Fig. 1:**
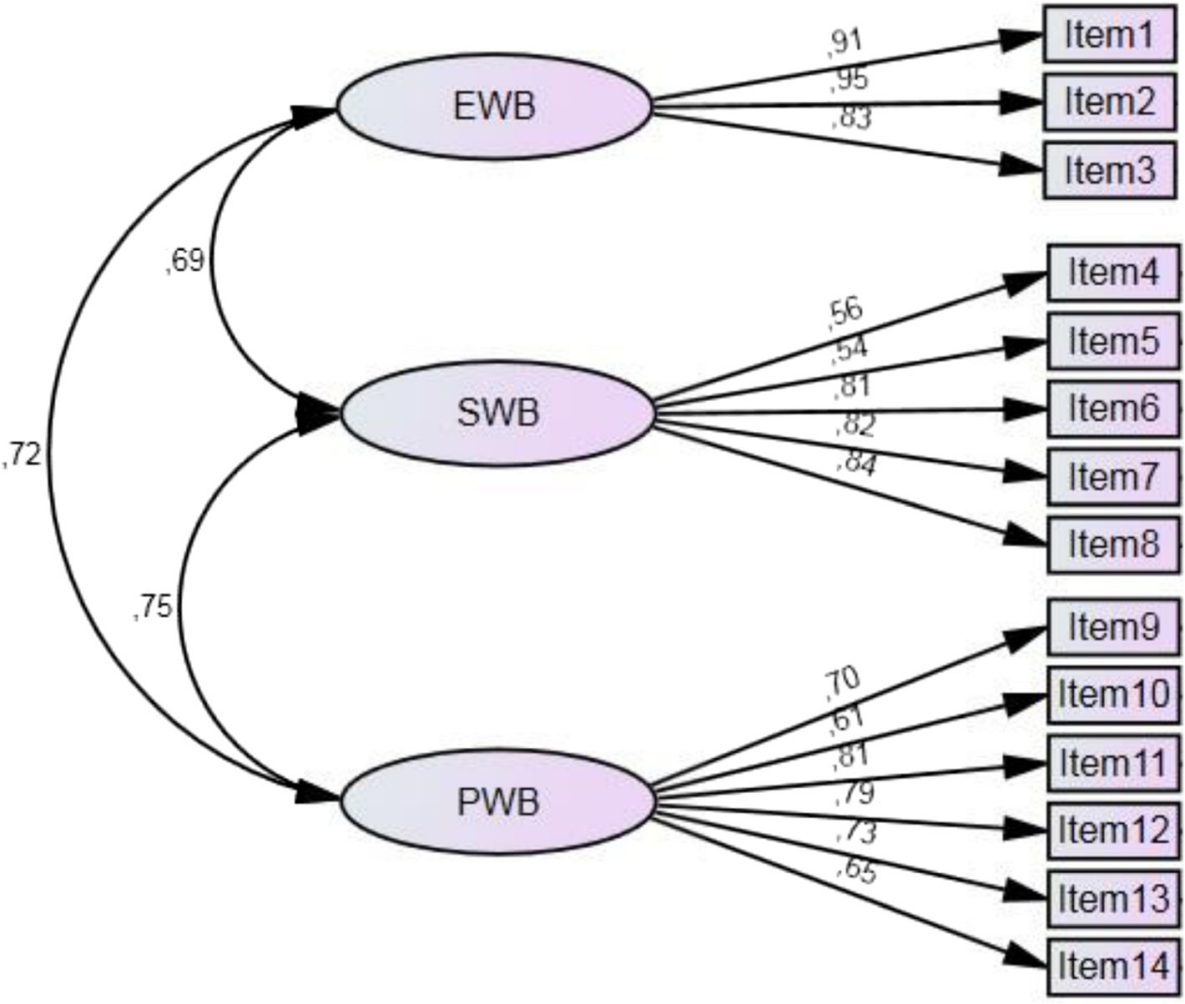
Confirmatory factor analysis of the three-factor model of MHC-SF

Three types of conceptual models were tested according to theoretical consideration and previous studies: (a) a single factor model only presenting mental health; (b) a dual factor model comprising one latent factor presenting hedonic well-being and one factor presenting eudaimonic well-being; (c) a triple factor model based on our hypothesis. Table 1 shows the results of confirmatory factor analysis for each model. The fit indices of the one-factor and two-factor models indicated poor fit of model overall. The root mean square error of approximation suggested the best fit of the three-factor model, while the values of the other two models had far from acceptable cutoff points. Other fit indices also indicated that the three-factor model has better model fits than its counterparts. Figure 1 presents the results of the confirmatory factor analysis for the three-factor model. The three-dimensional structure including emotional, psychological, and social well-being fits the data in all samples. Full configural and metric invariance was confirmed across grade and gender. The whole sample was distinguished into two grade groups from middle schools and high schools, respectively. The results are shown in Table 2.

**Table 1:** Maximum Likelihood Estimation of CFA Models of the Latent Structure of the MHC-SF terms

Fit indices	One factor	Two factors	Three factors
*Df*	77	76	74
RMSEA	0.159	0.117	0.086
CFI	0.775	0.878	0.936
Standardized RMR	0.073	0.059	0.057
IFI	0.775	0.878	0.936

*df* degree of freedom, *RMSEA* root mean square error of approximation, *CFI* comparative fit index; Standardized, *RMR* standardized root mean square residual, *IFI* incremental fit index, *CFA* confirmatory factor analysis, *MHC-SF* Mental Health Continuum-Short Form

**Table 2:** Measurement invariance of three dimensions of the MHC-SF across gender and age groups

	Model fit indices					
	Chi-square	*df*	CFI	RMSEA	SRMR	∆CFI
*Gender groups*						
Configural invariance	2916.208	148	0.937	0.061	0.056	
Metric invariance	3008.571	162	0.935	0.059	0.070	−0.002
*Grades*						
Configural invariance	3106.890	148	0.935	0.061	0.055	
Metric invariance	3189.232	162	0.933	0.059	0.063	−0.002

*df* degree of freedom, *RMSEA* root mean square error of approximation, *CFI* comparative fit index; Standardized, *RMR* standardized root mean square residual, *MHC-SF* Mental Health Continuum-Short Form

### External validity

The correlations of MHC-SF subscales with subscales and total scales of MMQL and HADS are presented in Table 3. All correlations were statistically significant. All MHC-SF scales correlated positively with the subscales of MMQL. The strongest correlations were found for emotional well-being and psychological well-being while the weakest correlation was seen for social well-being. Furthermore, the subscales of anxiety and depression both correlated with MHC-SF negatively. The strongest single correlation was a positive one and found between the total scale of MHC-SF and the total scale of MMQL (0.642). The strongest negative correlation found between the total scale of MHC-SF and the total scale of HADS was as expected noticeably weaker (−0.482), the difference was statistically significant (Δz >1.96).

**Table 3:** Bivariate correlations of MHC-SF and MMQL and HADS scales

	Emotional well-being	Social well-being	Psychological well-being	MHC-SF total scale
MMQL				
Physical functioning	0.418	0.396	0.438	0.468
Cognitive functioning	0.375	0.362	0.446	0.448
Psychological functioning	0.437	0.375	0.411	0.453
Body image	0.341	0.320	0.400	0.399
Social functioning	0.458	0.464	0.525	0.548
Outlook on life	0.525	0.491	0.520	0.573
Intimate relations	0.380	0.353	0.431	0.436
MMQL total scale	0.567	0.529	0.614	0.642
HADS				
Anxiety	−0.393	−0.344	−0.383	−0.418
Depression	−0.343	−0.326	−0.338	−0.376
HADS total scale	−0.465	−0.394	−0.427	−0.482

All correlations are significant at the .01 level (two-tailed)

The strength of all correlations between the Social Well-being dimension of MHC-SF and the subscales of MMQL and HADS were statistically significantly weaker (*p* < 0.01) as compared to the correlations between the Emotional and Psychological Well-being dimensions of MHC-SF and the subscales of MMQL and HADS, except ‘Depression’

### Floor and ceiling effects

The number and percentage of participants in each subgroup diagnosed with languishing, moderately mentally healthy and flourishing is shown in Table 4.

**Table 4:** Prevalence of mental health by subgroups

	Categorical diagnosis		
	Mentally unhealthy, languishing	Moderately mentally healthy	Mentally healthy, flourishing
Total	283 (5.2 %)	2019 (37.4 %)	3097 (57.4 %)
Grade			
8	180 (5.9 %)	1056 (34.7 %)	1808 (59.4 %)
10	103 (4.4 %)	963 (40.9 %)	1289 (54.7 %)
Gender			
Female	118 (4.3 %)	1042 (37.8 %)	1597 (57.9 %)
Male	157 (6.4 %)	905 (37.1 %)	1375 (56.4 %)

Table 5 presents the percentage of participants rating the highest and lowest scales for each dimension of MHC-SF. Floor effects were negligible for all items (2 %-14 %) while substantial ceiling effects were observed except Item 4 (30 %-40 %). Table 6 presents the percentage of participants scoring at the floor and ceiling of total and subscales for each subgroup. Notable ceiling effects for EWB occurred for all subgroups (20 %-29 %).

**Table 5:** Distribution in percentage on the lowest and highest rating scale for the items

Item	Label	Lowest Rating	Highest Rating
Item 1	Happiness, joy	1.5 %	30.8 %
Item 2	Interested in life	1.7 %	34.1 %
Item 3	Content/satisfied	3.0 %	30.5 %
Item 4	That you have something important to contribute to the society	8.9 %	12.9 %
Item 5	That you belong to a community	13.9 %	33.9 %
Item 6	That our society is becoming a better place for all people	8.2 %	31.7 %
Item 7	That people are basically good	5.7 %	32.6 %
Item 8	That the way society works is logical	5.3 %	32.5 %
Item 9	That you like most of your personality	3.3 %	39.8 %
Item10	That you are good at managing responsibility for your daily life	3.0 %	36.2 %
Item11	That life has a purpose	2.9 %	36.9 %
Item12	That you have warm and confident relationships with others	2.8 %	37.1 %
Item13	That you experience things that will make you grow as a person	3.6 %	29.4 %
Item14	That you have the confidence to have your own thoughts and that you dare to express them	4.1 %	32.1 %

**Table 6:** Scale floor and ceiling effects on total and sub-scales by subgroups

	EWB		SWB		PWB		Total score	
	% Floor	% Ceiling	% Floor	% Ceiling	% Floor	% Ceiling	% Floor	% Ceiling
Total	1.1	24.7	1.4	8.3	0.7	11.9	0.6	5.3
Age								
Grade 8	1.2	28.7	1.4	10.3	0.7	13.7	0.5	6.6
Grade 10	0.9	19.2	1.2	5.8	0.5	9.5	0.5	3.5
Gender								
Female	0.4	22.7	0.7	6.5	0.3	10.2	0.2	3.9
Male	1.8	26.9	2.1	10.3	1.1	13.9	1.0	6.8

## Discussion

This is to the best of our knowledge the first time that the psychometric properties of the MHC-SF are being evaluated on Chinese adolescents. The findings contribute to the global use of this instrument and enable comparisons between countries and cultures. In line with modern definitions of mental health as something more than merely the absence of mental illness, this instrument was developed to measure and to categorize positive mental health. In particular it was designed to be used in epidemiological monitoring, i.e., public health surveys.

High internal consistency was found in our study for the total scale of MHC-SF as well as the subscales, which is in line with other recent studies. Excellent internal consistency (> .80) of MHC-SF in adolescents [20, 21] and adults [21–24, 26–28] was also demonstrated in other countries, for example, Denmark, India, Italy, South Korea and United States. Confirmatory factor analysis yielded support for the three-dimensional factor model with results much in line with recent European, Asian and American studies [20–28, 47]. Our results are similar with the previous studies on adolescents with CFI greater than 0.90 [20, 21, 25] and SRMR less than 0.10 [21, 25]. Additionally, our findings supported the full configural invariance and metric invariance of the three subscales of the MHC-SF by gender and age group.

Floor effects for all subscales of MHC-SF were negligible while substantial ceiling effects were found for the emotional well-being subscale. The latter is of course related to the number of items (only 3) in that scale since ceiling effects per single item of the emotional well-being subscale were not any more pronounced than items of the other subscales. Thus, if the researcher’s interest lies primarily in the upper end of the MHC-SF scale ceiling effects could be problematic particularly for respondents in Grade 8, as ceiling effects were more pronounced among the youngest. However, in epidemiological surveillance and primary prevention the main interest would not be at the very top of the scale – at least not at this early stage of research on the concept of flouring. Nevertheless, the association between grade and ceiling effects in the present study should be kept in mind when descriptive results are compared with another Asian study [25]. The present study comprised of mostly 15 year-olds and 57, 4 % of them were categorized as mentally flourishing.

Regarding external validity positive correlations were found between MHC-SF subscales and relevant MMQL subscales as well as the total scale. In addition, negative correlations were found between MHC-SF and HADS subscales and the total scale. The stronger correlation found between MHC-SF and Minneapolis-Manchester Quality of Life Instrument than between MHC-SF and Hospital Anxiety Depression Scale yielded support for external validity. Previous studies have also shown support for external validity of the MHC-SF by correlations between MHC-SF and anxiety and depression [20–27] as well as health-related quality of life [20, 22–24, 26].

Further, in the present study, it should be noted that the strength of all correlations (*p* < 0.01) between the social well-being dimension of MHC-SF and the subscales of MMQL and HADS were statistically significantly weaker as compared to the correlations between the emotional and psychological well-being dimensions of MHC-SF and subscales of MMQL and HADS, except with the subscale of ‘Depression’. We suggest that these differences in the strength of observed associations are related to the theoretical distinction of emotional, psychological- and social well-being. For example, emotional well-being reflects the presence of positive feelings about life while psychological well-being incorporates the dimensions of self-acceptance, positive relations with others, personal growth, purpose in life environmental mastery and autonomy of the individuals. Social well-being, however, emphasizes social challenges and tasks as public criteria [17].

Some limitations of this study should be noted. Firstly, the assessment of external validity is the weaker part of the study, mainly because we did not include another scale of mental well-being like in another study [21]. On the other hand, such scales validated on Chinese adolescents are rare – if they exist. Instead we used scales for depression and anxiety as a measure of distress and illness, which have been shown previously to correlate negatively with MHC-SF [20–27], and health-related quality of life as a positive measure, which gives information on the individual’s whole life situation and its relation to mental well-being. This study is one part of a comprehensive research on life and health of adolescents emphasizing mental health. The questionnaire comprised of several scales and in total some 220 items about different dimensions of life and health in total. Considering the time limit for students to fill in the questionnaire, researchers restricted the number of items and instruments to be included in the questionnaire, i.e., more comprehensive assessments of mental well-being and mental illnesses. Also we should mention that although HADS is a widely used instrument [36–39] and has been psychometrically evaluated on Chinese adolescents [39], the latter version of the instrument was developed in a Hong Kong context in a different variant of the Chinese mandarin language than the one used in the current study. Therefore we carried out pre-testing of the instruments on 285 students in Weifang prior to the current study was performed. This pilot study helped us with improving face validity by adjusting the wording in some of the survey items.

Secondly, we were concerned with if the two different ways of collecting the questionnaire data would also have impacted the main results. Due to the high authority of some of the schoolmasters, students from three of the seven schools had to complete the questionnaire at home instead of in school and return it to the school teachers the following day. Hence, our concern was that those respondents could have been influenced by their peers and parents when filling in the questionnaires. For example, they might have indicated better mental health than otherwise in order not to have the information that they voluntarily provided made public or used against them [48]. In terms of factor structure of the MHC-SF there was no statistically significant difference since additional CFA confirmed the full configural and metric invariance across the two groups (∆CFI =0.001, ∆RMSEA = 0.003, ∆SRMR = 0.005). To analyze differences in the other results, i.e., correlations between scales, floor and ceiling effects and descriptive results on the categorization of positive mental health is more complicated because the method of collection was also associated with age. All participants from grade 10 filled in the questionnaires at home whereas most participants from grade 8 completed the questionnaire at school. Thus any such significant differences between the groups could be confounded by age.

## Conclusion

The findings of the present study offer evidence to support the use of the MHC-SF on adolescents and are in line with the studies from other countries that evaluated the psychometric properties of the MHC-SF. The findings suggest that MHC-SF is a useful instrument in assessment of adolescent mental health in China. As a result, the study contributes in filling the knowledge gap on the validation and usefulness of MHC-SF in national cultures world-wide to measure positive mental health.
