# Erratum to: psychometric evaluation of the Mental Health Continuum-Short Form (MHC-SF) in Chinese adolescents – a methodological study

**DOI:** 10.1186/s12955-015-0404-4

**Published:** 2016-01-07

**Authors:** Cheng Guo, Göran Tomson, Jizhi Guo, Xiangyun Li, Christina Keller, Fredrik Söderqvist

**Affiliations:** Medical Management Centre, Department of Learning, Informatics, Management and Ethics, Karolinska Institutet, Stockholm, Sweden; Department of Public Health Sciences, Karolinska Institutet, Stockholm, Sweden; School of Management, Weifang Medical University, Weifang, China; Jönköping University, International Business School, Jönköping, Sweden; Center for Clinical Research, Uppsala University, County hospital, Västerås, Sweden; Competence Center for Health, County Council of Västmanland, Västerås Hospital, Västerås, Sweden

Unfortunately, the original version of this article [1] contained two mistakes. The text on page 5 reading, “Floor effects were negligible for all items (2 %–14 %) while substantial ceiling effects were observed except Item 4 (30 %–40 %)” should instead have read, “Floor effects were negligible (2 %–14 %) while substantial ceiling effects were observed for all items (30 %–40 %) except Item 4”.

In Table 5 (Table 1 here), there is a mistake in the ordering of items. The corrected table can be seen below.

**Table 1:** Distribution in percentage on the lowest and highest rating scale for the items

Item	Label	Lowest rating	Highest rating
Item 1	Happiness, joy	1.5 %	30.8 %
Item 2	Interested in life	1.7 %	34.1 %
Item 3	Content/satisfied	3.0 %	30.5 %
Item 4	That you have something important to contribute to the society	8.9 %	12.9 %
Item 5	That you belong to a community	13.9 %	33.9 %
Item 6	That our society is becoming a better place for all people	8.2 %	31.7 %
Item 7	That people are basically good	5.7 %	32.6 %
Item 8	That the way society works is logical	5.3 %	32.5 %
Item 9	That you like most of your personality	3.3 %	39.8 %
Item 10	That you are good at managing responsibility for your daily life	3.0 %	36.2 %
Item11	That you have warm and confident relationships with others	2.8 %	37.1 %
Item12	That you experience things that will make you grow as a person	3.6 %	29.4 %
Item13	That you have the confidence to have your own thoughts and that you dare to express them	4.1 %	32.1 %
Item14	That life has a purpose	2.9 %	36.9 %
